# Modern Strategies for Diagnosis and Treatment of Parasitic Diseases

**DOI:** 10.3390/ijms25126373

**Published:** 2024-06-09

**Authors:** Leszek Rolbiecki, Joanna N. Izdebska

**Affiliations:** Department of Invertebrate Zoology and Parasitology, Faculty of Biology, University of Gdańsk, 80-308 Gdańsk, Poland; joanna.izdebska@ug.edu.pl

Parasites are very widely distributed in the environment and form complex relationships with their hosts, forming host–parasite systems. They have various life strategies, either parasitizing on the body of the host (ectoparasites) or inside it (endoparasites). They can form both permanent, often long-term, and temporary relationships and can demonstrate varying degrees of host specificity, from specialists to generalists. They often have complicated, complex life cycles characterized by various host strategies, which are selected at different stages of development. Moreover, their structure and life functions can range from microscopic, single-celled protozoans and multicellular, relatively primitive helminths to parasitic arthropods, complex animals with a nervous system demonstrating a highly advanced structure and functions [1,2,3,4].

Our understanding of a particular group of parasites depends to a great degree on the nature of the group and its region. Undoubtedly, parasites known to be pathogenic towards humans, pets, and farm/breeding animals are of greater interest; however, relatively little is known about these parasites. They function in an environment that is continually changing as a result of anthropogenic pressure and climate change and is subject to the influence of a range of complex conditions, e.g., in a wide range of reservoir hosts and their expanding geographical ranges. They can also become resistant to previously used treatment methods. Studies also indicate that their diversity is most likely much greater than currently recognized, as new species continue to be discovered and described and the introduction of molecular testing has revealed the presence of so-called cryptic species, which are difficult or impossible to identify based on morphoanatomical methods [5,6,7,8,9,10,11,12,13,14,15,16,17,18,19].

Therefore, there is still a lack of effective methods for detecting many parasites and for diagnosing and treating parasitoses and their related symptoms, such as allergic reactions and pathogen transmission. There is also a need to improve and develop parasitological monitoring and forecasting methods in response to local and global environmental changes, such as climate change, which often expand the range of the host and thus the parasite [20,21,22,23,24,25].

It is hence essential to improve our research approaches with the support of modern techniques; one such useful, even indispensable, armamentarium for parasitological research, and the standard in some areas, comprises methods based on molecular biology. Such approaches are increasingly used in the taxonomic identification of parasites, especially microscopic organisms that are difficult to identify, or parasites with evolutionary modifications which can obfuscate their rank and systematic position. Such analyses are of great importance for the study of biodiversity, geographical spread, and pathogen transmission, and for determining relationships and analyzing dependencies in complex host–parasite systems. Molecular methods are an indispensable tool within the spectrum of advanced diagnostic methods (especially in the context of hard-to-detect parasites) and therapeutic methods (such as drug and vaccine design) [26,27,28,29,30,31,32,33,34,35,36,37,38,39,40,41,42,43,44,45,46]. As such, researchers must develop diverse, unique approaches to various research topics, depending on the group being studied, the scientific problem, the scope and area of research, and their specific goals.

The current Special Issue has therefore become not only a collection that shows the diverse nature of the research issues in contemporary parasitology, but also a platform for discussion on the possibilities of using various methods from the field of molecular biology [47,48,49,50,51,52,53,54]. The area has recently been subject to an enormous growth in research and, as such, its status requires constant updating and specialized verification; hence, review studies critically analyzing the current state of the knowledge in this area are also invaluable.

Molecular methods have revolutionized a number of parasitological specialisms, including the study of single-celled organisms, which were previously difficult to detect and identify due to their microscopic size and the small range of features they have that are useful in taxonomic studies based on morphoanatomy [47,48,49,50,51,52,53]. These methods can be used in a wide spectrum of research, such as population studies aiming to detect parasites and estimate the frequency of their occurrence in various human populations and communities, thus monitoring changes and detecting new threats [47]. One such study employed molecular methods to detect and identify *Blastocystis* sp. in Polish soldiers stationed in the Republic of Kosovo. Research has shown that a longer stay in a new environment can result in a significant increase in infection; these findings may further the development of prevention procedures [47]. In terms of environmental monitoring, research related to the detection of parasites in various reservoir hosts is also important. One such important, although poorly understood, parasitological group comprises bats, which may be a source of parasites with zoonotic potential [48]. Thus, in a study of three species of common and widely distributed bats, *Toxoplasma gondii*, *Neospora caninum*, and *Encephalitozoon* spp. microsporidia DNA was detected; this study offers new data in this field, not only on the European scale, but also on the global scale. It also confirms the role of bats as reservoir hosts of parasites important to humans and the animals associated with them [48].

Expanding and extending the range of molecular tools available to us is of great importance for parasitological diagnostics and may also change our therapeutic approach to parasites [49,50,51,52]. This may include, for example, new opportunities to use improved, more effective diagnostic tests for common and often dangerous intestinal parasites that cause disease and death, such as *Cryptosporidium* spp., *Giardia intestinalis*, and *Entamoeba histolytica* [49]. In turn, research conducted around the world, based on a new approach using a combination of genomics and proteomics, has significantly contributed to the development of new diagnostic tools, vaccines, and drugs for cryptosporidiosis [50]. Another modern approach that employs molecular research tools has also allowed for the development of innovative diagnostic methods for detecting human amoebiasis, especially the identification, genomic analysis, and examination of the genetic diversity of *Entamoeba* spp. and the association of specific genotypes with various forms of human amoebiasis [51].

The development of research methods and new therapeutic approaches is clearly visible in a study of the pathogenic protozoa *Trypanosoma* spp. [52,53]. This study’s findings indicate that cyclophilin (CyP) enzymes can be used as early biomarkers of the effectiveness of a treatment for trypanosomiasis (Chagas disease), which is caused by *Trypanosoma cruzi* [52]. Similarly, the introduction of modern diagnostic methods has allowed for the timely and accurate diagnosis of human African trypanosomiasis caused by *T. brucei*. This early intervention is crucial for its effective treatment, and the introduction of new therapies may contribute to the complete elimination of this disease in the near future [53].

Modern methods based on integrative approaches that combine achievements and tools from various specialties have also fueled the development of scientific research on multicellular parasites; these approaches have generated improvements in diagnostic methods and led to the introduction of new therapies useful in medicine and veterinary medicine. An example is the study of parasitic nematodes in the gastrointestinal tract of small ruminants, which cause significant losses on animal farms. Research, and especially immunological research, is employed in the search for new strategies to prevent parasitosis and develop antiparasitic vaccines [54].

While the scope of the possibilities associated with the modern parasitological research methods presented herein will undoubtedly not exhaust the topic, they will hopefully serve as a source of inspiration for new ideas.
